# Adenosine A_2B_ receptor-mediated leukemia inhibitory factor release from astrocytes protects cortical neurons against excitotoxicity

**DOI:** 10.1186/1742-2094-9-198

**Published:** 2012-08-16

**Authors:** Shamsudheen Moidunny, Jonathan Vinet, Evelyn Wesseling, Johan Bijzet, Chu-Hsin Shieh, Sven CD van Ijzendoorn, Paola Bezzi, Hendrikus WGM Boddeke, Knut Biber

**Affiliations:** 1 Department of Neuroscience, Section Medical Physiology, University Medical Center Groningen, University of Groningen, A. Deusinglaan 1, 9713 AV, Groningen, The Netherlands; 2 Department of Rheumatology and Clinical Immunology, University Medical Center Groningen, University of Groningen, A. Deusinglaan 1, 9713 AV, Groningen, The Netherlands; 3 Department of Psychiatry and Psychotherapy, Section Molecular Psychiatry, University of Freiburg, Hauptstrasse 5, 79104, Freiburg, Germany; 4 Department of Cell Biology, Section Membrane Cell Biology, University Medical Center Groningen, University of Groningen, A. Deusinglaan 1, 9713 AV, Groningen, The Netherlands; 5 Department of Cell Biology and Morphology, University of Lausanne, Rue du Bugnon 9, 1005, Lausanne, Switzerland

**Keywords:** 5′-N-Ethylcarboxamide (NECA), Leukemia inhibitory factor, Neuroprotection, Glutamate

## Abstract

**Background:**

Neuroprotective and neurotrophic properties of leukemia inhibitory factor (LIF) have been widely reported. In the central nervous system (CNS), astrocytes are the major source for LIF, expression of which is enhanced following disturbances leading to neuronal damage. How astrocytic LIF expression is regulated, however, has remained an unanswered question. Since neuronal stress is associated with production of extracellular adenosine, we investigated whether LIF expression in astrocytes was mediated through adenosine receptor signaling.

**Methods:**

Mouse cortical neuronal and astrocyte cultures from wild-type and adenosine A_2B_ receptor knock-out animals, as well as adenosine receptor agonists/antagonists and various enzymatic inhibitors, were used to study LIF expression and release in astrocytes. When needed, a one-way analysis of variance (ANOVA) followed by Bonferroni post-hoc test was used for statistical analysis.

**Results:**

We show here that glutamate-stressed cortical neurons induce LIF expression through activation of adenosine A_2B_ receptor subtype in cultured astrocytes and require signaling of protein kinase C (PKC), mitogen-activated protein kinases (MAPKs: p38 and ERK1/2), and the nuclear transcription factor (NF)-κB. Moreover, LIF concentration in the supernatant in response to 5′-N-ethylcarboxamide (NECA) stimulation was directly correlated to *de novo* protein synthesis, suggesting that LIF release did not occur through a regulated release pathway. Immunocytochemistry experiments show that LIF-containing vesicles co-localize with clathrin and Rab11, but not with pHogrin, Chromogranin (Cg)A and CgB, suggesting that LIF might be secreted through recycling endosomes. We further show that pre-treatment with supernatants from NECA-treated astrocytes increased survival of cultured cortical neurons against glutamate, which was absent when the supernatants were pre-treated with an anti-LIF neutralizing antibody.

**Conclusions:**

Adenosine from glutamate-stressed neurons induces rapid LIF release in astrocytes. This rapid release of LIF promotes the survival of cortical neurons against excitotoxicity.

## Background

Leukemia inhibitory factor (LIF) is a soluble glycoprotein that belongs to the family of interleukin (IL)-6-type cytokines. Other members of this family include IL-6, IL-11, ciliary neurotrophic factor (CNTF), oncostatin M (OSM), cardiotrophin-1 (CT-1) and novel neurotrophin-1 (NNT-1) [1], which display pronounced trophic as well as protective properties during pathophysiology of the central nervous system (CNS) and are hence referred to as neuropoietic cytokines or neurokines [2]. Specific functions of LIF in the nervous system include induction of cholinergic differentiation of sympathetic neurons, induction of neuropeptide and choline acetyltransferase (ChAT) gene expression [3], regulation of polyneuronal innervation of neuromuscular junction [4,5] and regulation of the HPA axis [6,7]. Furthermore, LIF signaling is crucial for development of the nervous system, including development of sensory and motor neurons [8,9] and glial cells [10]. Consistently, reduced numbers of astrocytes and oligodendrocytes are found in LIF knock-out mice [11]. During inflammation, LIF has been suggested to be both pro- and anti-inflammatory and appears to play a key role in neural injury and regeneration. We and others have previously demonstrated the neuroprotective properties of LIF against damages caused by excitotoxicity, light, *et cetera*[12-14]. Moreover, promotion of axonal regeneration and oligodendrocyte growth and survival by LIF suggests its potential for reducing damage associated with central inflammatory demyelinating diseases such as multiple sclerosis [15-17].

In the CNS, astrocytes are considered to be the major source for LIF [18,19], and its expression in the brain is significant during pathological conditions including ischemia [20,21], multiple sclerosis [22], Alzheimer’s and Parkinson’s diseases [23] and brain injury [24]. The factors responsible for elevated LIF induction during CNS pathology are largely unknown. One of the candidates identified recently to induce LIF expression in astrocytes is ATP [18,25], levels of which also rise during conditions like high-frequency neuronal activity, seizure, ischemia and hypoxia [26,27]. However, extracellular ATP is rapidly hydrolyzed by a cascade of ectonucleotidases resulting in an enhanced level of adenosine [26,28]. Correspondingly, excitotoxic conditions such as ischemia, hypoxia, seizure and head injury are known to induce a rapid increase in extracellular adenosine concentrations, up to 100 times that of the resting concentration [29-33]. There is abundant evidence for immune regulation by adenosine [34] including expression and release of growth factors and cytokines such as nerve growth factor (NGF), S100beta, IL-6 and CCL2 in glial cells [35-39]. However, it is not known whether adenosine can induce LIF expression in astrocytes.

In the present study, we investigated the potential influence of adenosine receptor activity on LIF release from cultured astrocytes.

## Methods

### Chemicals and reagents

Neurobasal media, Hank’s balanced salt solution (HBSS), phosphate-buffered saline (PBS), sodium pyruvate, L-glutamine, penicillin-streptomycin, hydroxyethyl piperazineethanesulfonic acid (HEPES), glutaMAX-1 and B27 supplement were obtained from Gibco (Breda, The Netherlands). Dulbecco’s modified Eagle’s medium (DMEM) and fetal calf serum (FCS) were obtained from PAA Laboratories (Cölbe, Germany). Trypsin was obtained from Life Technologies (Breda, The Netherlands). L-leucine methyl ester (LME) and the remaining cell medium components were purchased from Sigma-Aldrich (Zwijndrecht, The Netherlands). Recombinant mouse LIF (rmLIF: LIF2005) was obtained from Millipore (Amsterdam, The Netherlands). Brefeldin A (BFA), caffeine, L-glutamate, adenosine A_2B_ receptor antagonist (MRS 1754), protein kinase A (PKA) inhibitor (KT 5720), protein kinase C (PKC) inhibitor (Ro 31–8220), p38 mitogen-activated protein kinases (MAPK) inhibitor (SB 203580), and adenosine analog (5′-N-Ethylcarboxamide or NECA) were obtained from Sigma-Aldrich (Zwijndrecht, The Netherlands). Non-hydrolysable ATP (2MeSATP), adenosine A_2A_ receptor antagonist (ZM 241385), adenosine A_2A_ receptor agonist (CGS 21680) and MEK1/2 inhibitor (U 0126) were obtained from Tocris Bioscience (Bristol, UK). NF-kB inhibitor (BAY 11–7082) and c-Jun N-terminal kinase (JNK) inhibitor (SP 600125) were obtained from Calbiochem (Darmstadt, Germany). Reagents used in immunoblotting experiments were purchased from Bio-Rad Laboratories (Veenendaal, The Netherlands) with the exception of the polyvinylidene fluoride (PVDF) membranes that were obtained from Millipore (Bedford, MA).

### Animals

Wild-type C57BL/6 J (1 to 2 days postnatal) mice were obtained from Central Laboratory Animal Facility (University of Groningen, The Netherlands). Adenosine A_2B_ receptor knock-out (A_2B_ KO) mice (1 to 2 days postnatal) with the same genetic background were kindly provided by Professor Marco Idzko (University of Freiburg, Germany). Wild-type C57BL/6 J (14 to 15 days embryonic) mice were obtained from Harlan (Horst, The Netherlands). All procedures were in accordance with the regulation of the Ethical Committee for the use of experimental animals of the University of Groningen, The Netherlands (License number DEC 4623A and DEC 5913A). Animals were housed in standard Makrolon™^TM^ (Bayer AG, Leverkusen, Germany) cages and maintained on a 12 hour light/dark cycle. They received food and water *ad libitum*.

### Primary neuronal culture

Primary culture of cortical neurons from mouse embryo (~E_15_) was established as described previously [13]. Briefly, cortices from embryonic brains were dissected in ice-cold HBSS supplemented with 30% glucose. Meninges were removed, and the tissues were treated with trypsin before they were gently dissociated by trituration in neuronal culture media (neurobasal medium supplemented with 2% B27, 1 mM sodium pyruvate, 2 mM L-glutamine and 50 U/mL penicillin-streptomycin). The cell suspension was filtered using cell strainer (70 μm) (BD Falcon, Franklin Lakes, NJ, USA) before centrifugation (800 rpm for 10 minutes). Cells were then seeded on poly-D-lysine-coated six-well plates (1.5 x 10^6^ cells/well) and maintained in neuronal culture media in a humidified atmosphere with 5% CO_2_ at 37°C. The culture medium was refreshed the next day to get rid of debris. The neuronal purity as determined by Microtubule-associated protein 2 (MAP2)-staining was around 98% (data not shown) [13]. Cultures were used after 5 days *in vitro*.

### Induction of excitotoxicity

Cortical neuron cultures were subjected to an excitotoxic challenge with glutamate (50 μM, for 1 hour), after which cultures were refreshed with fresh media and were incubated at 37°C. Supernatants from neuron cultures (untreated and glutamate-treated) were collected 18 hours after glutamate challenge and were applied to the primary astrocyte cultures.

### Primary astrocyte cultures

Primary astrocyte cultures were established from cerebral cortices of postnatal (1 to 2 days) C57BL/6 J and A_2B_ KO mice according to a previously described procedure [40], which was modified to reduce microglial contamination [41]. Microglial cells were separated from the astrocytic monolayer by 1-hour shake-off at 150 rpm. This procedure was repeated two times with an interval of 4 days *in vitro* between each shake off, followed by an overnight shake-off at 240 rpm to remove oligodendrocyte precursor cells. Purified astrocytes were washed with HBSS buffer containing 1 mM ethylenediaminetetraacetic acid (EDTA) and further detached using HBSS with 0.1% trypsin. Cells were reseeded with fresh astrocyte culture medium (DMEM supplemented with 5% FCS, 2 mM L-glutamine, 1 mM sodium pyruvate and 50 U/mL penicillin-streptomycin) in multi-well plates (5 x 10^4^ cells/cm^2^) and maintained in culture to confluency. To further reduce microglial contamination, confluent astrocyte cultures were treated with 5 mM LME, a lysosomotropic agent [42], for 4 to 5 hours. Astrocytes were ready for experiments after 1 to 2 days. Our cell preparations had a high percentage of astrocytes (≥95%), which was confirmed by immunostaining against GFAP (astrocyte specific marker) and CD11b (microglial specific marker) (data not shown).

### Real-time polymerase chain reaction

Total RNA of primary astrocytes was extracted, purified and transcribed into cDNA as described previously [13]. Quality of the cDNA was examined using the following housekeeping gene *Glyceraldehyde-3-phosphate dehydrogenase* (*GAPDH*) primer pairs: Fw 5′-CATCCTGCACCACCAACTGCTTAG-3′ and Rev 5′-GCCTGCTTCACCACCTTCTTGATG-3′ [Accession number: NM-008084]. The effect of neuronal supernatants and NECA on LIF mRNA expression in cultured astrocytes was analyzed by real-time PCR (qPCR) using the iCycler and iQ™ SYBR™ Green supermix (Bio-Rad, Veenendaal, The Netherlands ). Mouse *Hypoxanthine phosphoribosyltransferase 1* (*HPRT1*) and *GAPDH* primers were used for normalization to housekeeping genes (data normalized to *HPRT1* are not shown), and these genes showed no variations in response to the experimental treatments. The primer pairs used for qPCR were: LIF (Fw 5′-ATGTGCGCCTAACATGACAG-3′ and Rev 5′-TATGCGACCATCCGATACAG-3′) [Accession number: NM-008501]; *GAPDH* (Fw 5′-ATGGCCTTCCGTGTTCCTAC-3′ and Rev 5′-GCCTGCTTCACCACCTTCTT-3′) [Accession number: AF106860] and *HPRT1* (Fw 5′-GACTTGCTCGAGATGTCA-3′ and Rev 5′-TGTAATCCAGCAGGTCAG-3′) [Accession number: NM-013556]. The comparative C_t_ method (amount of target amplicon X in Sample S, normalized to a reference R and related to a control sample C), was calculated by:

(1) $$2-\left(\left({\text{C}}_{\text{t}}\text{X},\text{S}-{\text{C}}_{\text{t}}\text{R},\text{S}\right)-\left({\text{C}}_{\text{t}}\text{X},\text{C}-{\text{C}}_{\text{t}}\text{R},\text{C}\right)\right)$$

and was used to determine the relative gene expression levels [43].

### Western blot

Western blotting on cultured cortical astrocytes was performed as previously described [13]. Equal amounts of protein (30 μg) were loaded to 12.5 or 15% sodium dodecyl sulfate-polyacrylamide gels and subsequently transferred to PVDF membranes. The membranes were blocked using Odyssey™ Blocking Buffer (OBB; LI-COR Biosciences, Cambridge, UK; diluted 1:1 in PBS) for 1 hour and incubated overnight at 4°C with different combinations of primary antibodies (diluted in 1:1 OBB and PBS + 0.1% Tween 20 (PBS-T)): mouse monoclonal anti-β-actin (1:8000, Abcam, Cambridge, UK); rabbit monoclonal anti-phospho-NF-κB p65 (Ser536) (1:1000, Cell Signaling Technology, Leiden, The Netherlands); and rat monoclonal anti-LIF (MAB449; 1 μg/mL, R&D Systems, Oxford, UK). The next day, membranes were washed in PBS-T (four times for 5 minutes each time) and incubated for 1 hour at room temperature with appropriate fluorescence conjugated secondary antibodies (diluted in PBS-T): donkey anti-mouse IR Dye 680 (1:10000, LI-COR Biosciences, Cambridge, UK); goat anti-rat IR Dye 680 (1:10000, LI-COR); and donkey anti-rabbit IR Dye 800CW (1:10000, LI-COR). Membranes were washed again in PBS-T (four times for 5 minutes each time) and the fluorescent bands were detected using LI-COR’s Odyssey™ infrared imaging system.

### Leukemia inhibitory factor ELISA

A total of 1 mL of supernatant was collected from each well of the six-well plates of primary mouse astrocyte cultures, and these samples were stored at −20°C. ELISA plates (96-well, Costar, Corning Life Sciences, Amsterdam, The Netherlands) were coated overnight at room temperature with 100 μl/well of primary antibody goat anti-LIF (AF449; 0.5 μg/mL, R&D Systems, Oxford, UK) diluted in 0.01 M PBS (pH 7.4). The following day, the plates were washed six times with wash buffer (0.25 M Tris–HCl pH 8, 0.15 M NaCl, 0.05% Tween-20) using an automated microplate washer and air dried (this step is repeated after each incubation step). Plates were subsequently incubated for 1 hour at room temperature with 200 μl/well of blocking buffer (0.01 M PBS, 2% BSA). After blocking, the plates were incubated with supernatants from astrocyte cultures (100 μl/well) for 2 hours at room temperature. Two dilutions (1:2 and 1:4) of each sample, diluted in incubation buffer (0.01 M PBS, 0.2% gelatin, 0.05% Tween-20), were made in triplicates. The plates were then incubated for 1 hour at room temperature with 100 μl/well of the detection antibody, biotinylated goat anti-LIF (BAF449; 0.05 μg/mL; R&D Systems, Oxford, UK) diluted in incubation buffer, followed by an incubation for 30 minutes at room temperature with 100 μl/well of Streptavidin-horseradish peroxidase (HRP) conjugate (1:8000, Sanquin Reagents, Amsterdam, The Netherlands). The plates were then incubated for 15 to 20 minutes at room temperature with 100 μl/well of TMB detection buffer (0.1 M acetate buffer, 0.1 M sodium-acetate, pH adjusted with 1 M citric acid (0.21 g/mL; dissolve 2 tablets of 3, 3′, 5, 5′-tetramethyl benzidinedihydrochloride in 11 mL of TMB buffer and add 2 μl of 30% H_2_O_2_)). Upon stable color formation the reactions were stopped by adding 100 μl/well of 1 M H_2_SO_4_. Absorbance of the samples was measured using VersaMax, a spectrophotometric ELISA plate reader, and SoftMax Pro software (Molecular Devices, CA, USA) at 450 nm, with a background correction at 575 nm. Recombinant mouse LIF (15 to 2000 pg/mL) was used to plot the standard curve.

### MTT assay

Survival of cultured embryonic cortical neurons or cultured neonatal astrocytes against various experimental treatments was measured by the colorimetric MTT (3-(4,5-dimethylthiazol-2-yl-) 2,5-diphenyltetrazolium bromide) assay, as described previously [44]. MTT solution (0.5 mg/mL final concentration) was added to cultured cells and incubated for 4 hours, after which, cells were lysed and MTT-formazan solubilized in dimethyl sulfoxide (DMSO) on an orbital shaker for 15 minutes. Optical density measure of each sample was determined using an automated ELISA reader - the Varioskan Flash spectral scanning multimode reader (Thermo Scientific, FL, USA) at 570 nm, with a background correction at 630 nm.

### Immunocytochemistry and confocal microscopy

Astrocytes cultured on glass cover slips were fixed for 15 minutes in 4% paraformaldehyde. After several washes in PBS, the cells were blocked for 45 minutes with 5% normal goat serum (Vector Laboratories, Burlingame, CA, USA) in PBS containing 0.1% TritonX (Sigma, Zwijndrecht, The Netherlands). The cover slips were then incubated overnight at 4°C with rat anti-LIF primary antibody (5 μg/mL, R&D Systems, Oxford, UK) in combination with one of the following primary antibodies: rabbit anti-Rab11 (1:400, Zymed, San Francisco, CA, USA); rabbit anti-chromogranin A & B (1:100, Novus Biologicals, Cambridge, UK); mouse anti-clathrin (1:1000, Abcam, Cambridge, UK); rabbit anti-pHogrin C-terminal (1:100, kind gift of Professor J.C. Hutton (Denver, USA)) and rabbit anti-giantin (1:1000, Covance, Princeton, NJ, USA). The following day, cells were rinsed three times with PBS and incubated for 1 hour with the appropriate secondary antibodies: donkey anti-rat CY3 (1:500, Jackson ImmunoResearch Laboratories, Uden, The Netherlands); donkey anti-rabbit Alexa Fluor 488 (1:500, Molecular Probes, Leiden, The Netherlands)and donkey anti-mouse Alexa Fluor 488 (1:500, Molecular Probes). The cover slips were then rinsed with PBS and mounted on microscopic slide with Mowiol (Sigma, Zwijndrecht, The Netherlands) and analyzed with a Leica SP2 AOBS system (Leica Microsystems, Rijswijk, The Netherlands). Pictures were deconvoluted using the software Huygens Pro (SVI, Hilversum, The Netherlands). Primary antibody omission served as the control.

### Statistical data analysis

The absolute data values were normalized to the control in order to allow multiple comparisons. Statistical analyses were performed by one-way analysis of variance (ANOVA) followed by Bonferroni post-hoc test, using the Statistical Package for the Social Sciences (SPSS, Chicago, IL, USA). In all cases, *P* values < 0.05 were considered statistically significant.

## Results

### Glutamate-challenged cortical neurons induce LIF expression in cultured astrocytes through adenosine receptor activation

**Figure 1:**
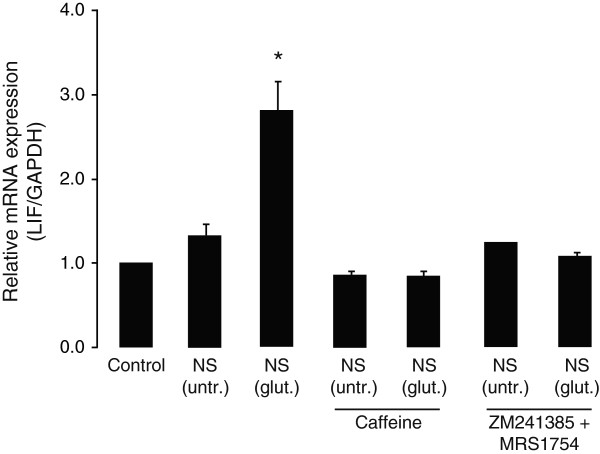
**Glutamate-stressed cortical neurons induce leukemia inhibitory factor (LIF) gene expression in primary cortical astrocytes, by a mechanism dependent on astrocytic adenosine receptor activation.** Supernatants of primary mouse cortical neurons (NS) collected 18 hours following treatment without (untr.) or with glutamate (glut.; 50 μM, for 1 hour) were applied (1:1 dilution by volume) to the primary cultured cortical astrocytes for 2 hours. Where indicated, astrocytes were pre-treated with caffeine (50 μM) or a cocktail of adenosine A_2A_ and A_2B_ receptor antagonists (A_2A_ antagonist: ZM 241385, 250 nM; A_2B_ antagonist: MRS 1754, 250 nM), for 30 minutes before incubation with neuronal supernatants and were analyzed for LIF mRNA expression (relative to *GAPDH*) using real-time PCR. Data are normalized to the control and presented as Mean ± SEM of three independent experiments. *P* < 0.05.

We have previously shown that treatment of cultured cortical neurons with glutamate (50 μM, for 1 hour) reduces cell survival by 60%, when compared to untreated controls [13]. In order to investigate whether neuronal death induces LIF synthesis in astrocytes, supernatants from cultured cortical neurons were collected 18 hours after glutamate treatment (50 μM for 1 hour) and applied to cultured cortical astrocytes. It is shown here that treatment of cultured astrocytes for 2 hours with supernatant from untreated neurons did not change LIF expression (Figure 1). On the other hand, supernatant from glutamate-challenged neurons induced approximately three times greater expression of astrocytic LIF mRNA (Figure 1). The induction of LIF mRNA expression by glutamate-challenged neuronal supernatants was absent in the presence of the non-specific adenosine receptor antagonist (caffeine, 50 μM) (Figure 1) and by cocktail of the specific adenosine A_2_ receptors antagonists (A_2A_ antagonist: ZM 241385, 250 nM; A_2B_ antagonist: MRS 1754, 250 nM) (Figure 1), suggesting that enhanced LIF expression in astrocytes induced by glutamate-challenged neuronal supernatants is mediated through adenosine A_2A_ and/or A_2B_ receptor subtypes. 

### NECA-induced LIF expression and secretion levels in cultured mouse astrocytes is concentration- and time-dependent

**Figure 2:**
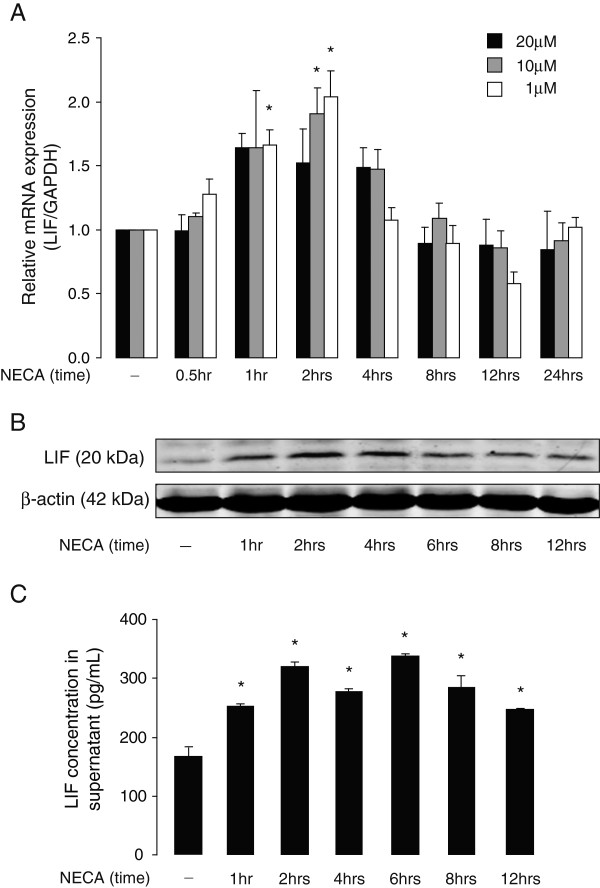
**NECA increases leukemia inhibitory factor (LIF) expression and secretion levels in primary mouse astrocytes. (A)** Primary cortical astrocytes were treated without or with NECA (1, 10 or 20 μM) for 0.5, 1, 2, 4, 8, 12 and 24 hours. Cells were then analyzed for LIF mRNA expression (relative to *GAPDH*) by real-time PCR. Data are normalized to the control and presented as Mean ± SEM of three independent experiments. *P* < 0.05. **(B)** shows Western blot analysis of cultured astrocytes treated without or with NECA (1 μM) for 1, 2, 4, 6, 8 and 12 hours to determine LIF protein levels. β-actin served as a loading control. **(C)** shows a representation of sandwich ELISA experiment performed to detect LIF content in supernatants of cultured astrocytes that were treated without or with NECA (1 μM) for 1, 2, 4, 6, 8 and 12 hours. Each bar corresponds to the mean concentration of LIF in triplicate samples; error bars indicate SEM. Observations were confirmed by repeating the experiments two additional times. *P* < 0.05. NECA, 5′-N-ethylcarboxamide.

In order to further investigate adenosine receptor-mediated LIF expression in astrocytes, we used NECA, a non-selective adenosine receptor agonist. As shown in Figure 2A, NECA-induced LIF mRNA expression in cultured astrocytes was concentration- and time-dependent, with maximum induction after 2 hours of incubation with 1 and 10 μM NECA. Subsequently, the effect of NECA (1 μM) on LIF protein expression was analyzed by Western blot. Elevated LIF protein expression was detected after 1 hour of NECA treatment, with a maximum induction after 2 to 4 hours (Figure 2B). Consistently, ELISA analysis revealed LIF protein content in supernatants from untreated astrocyte cultures, which increased in time upon treatment with NECA (1 μM) (Figure 2C).

### NECA-induced LIF expression and secretion levels is dependent on adenosine A_2B_ receptor activation

**Figure 3:**
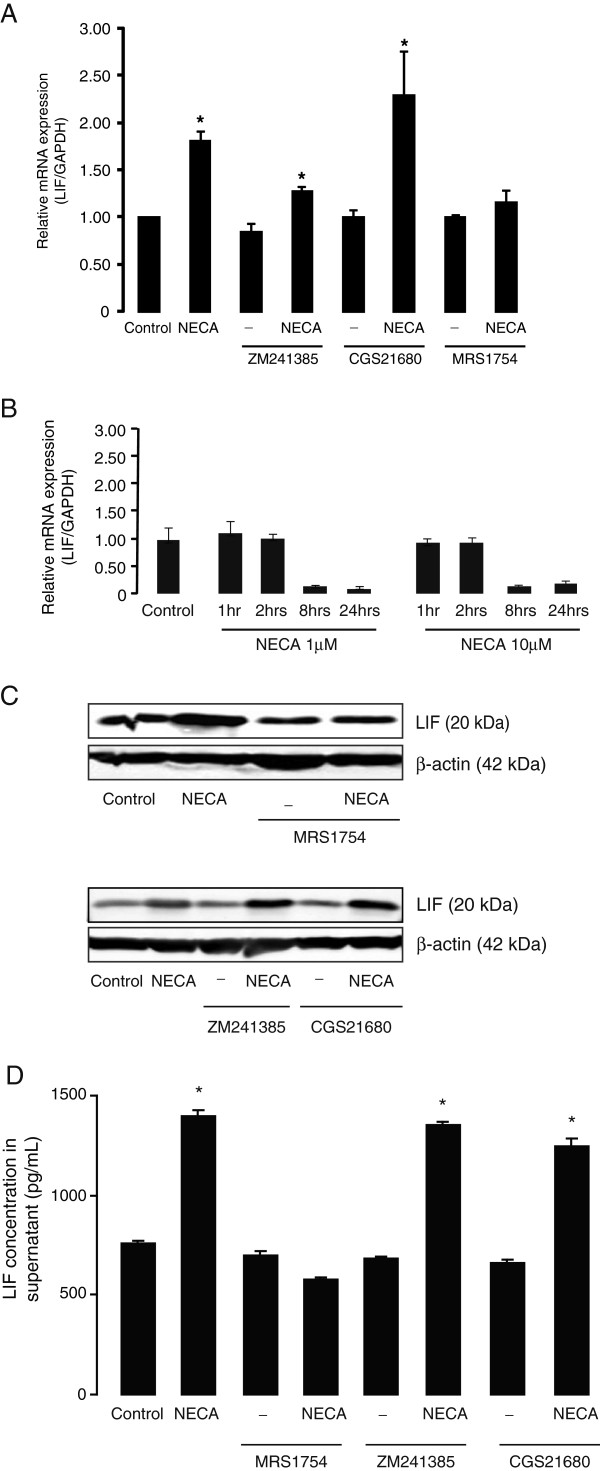
**NECA-induced leukemia inhibitory factor (LIF) expression and secretion levels in primary astrocytes are dependent on adenosine A**_**2B**_**receptor activation.** Primary cortical astrocytes were treated with the adenosine analog, NECA (1 μM) or a selective adenosine A_2A_ receptor agonist (CGS 21680, 1 μM) for 2 hours (for real-time PCR) or 4 hours (for Western blot and ELISA). Where indicated, astrocytes were pre-treated for 30 minutes with selective adenosine A_2A_ receptor antagonist (ZM 241385, 250 nM) or adenosine A_2B_ receptor antagonist (MRS 1754, 250 nM), prior to NECA stimulation. **(A and B)** show real-time PCR analyses of LIF gene expression (relative to *GAPDH*) in wild-type and A_2B_ knock-out astrocytes, respectively. Data are normalized to the control and presented as Mean ± SEM of three independent experiments. *P* < 0.05. **(C)** shows Western blot analyses to detect LIF protein levels in wild-type astrocytes. β-actin served as a loading control. **(D)** shows a representation of sandwich ELISA experiment performed to detect LIF content in supernatants of cultured wild-type astrocytes. Each bar corresponds to the mean concentration of LIF in triplicate samples; error bars indicate SEM. Observations were confirmed by repeating the experiments two additional times. *P* < 0.05. NECA, 5′-N-ethylcarboxamide.

In subsequent experiments, specific antagonists of adenosine A_2A_ and A_2B_ receptors (ZM 241385 and MRS 1754, respectively) were used to identify the receptor subtype involved in NECA-induced LIF expression and release in cultured astrocytes. Pre-treatment of astrocytes with ZM 241385 (250 nM, added 30 minutes before NECA) did not abolish NECA-induced LIF mRNA and protein expression (Figure 3A and 3C). In contrast, NECA-induced LIF mRNA (Figure 3A) and protein expression (Figure 3C) and release (Figure 3D) were completely inhibited by MRS 1754 pre-incubation (250 nM, added 30 minutes before NECA). In addition, specific adenosine A_2A_ receptor agonist (CGS 21680; 1 μM) failed to induce LIF mRNA or protein expression and release (Figure 3A, C, D). The involvement of A_2B_ receptors was further confirmed in A_2B_ KO astrocytes where NECA stimulation for up to 24 hours did not induce LIF mRNA expression (Figure 3B). Instead, astrocytes without A_2B_ receptors responded to NECA stimulation with a down-regulation of LIF mRNA at 8 and 24 hours (Figure 3B). Taken together, these results clearly show that NECA-induced LIF expression and release from cultured mouse astrocytes is dependent on the activation of adenosine A_2B_ receptors.

NECA-induced LIF expression and secretion levels in primary astrocytes are mediated through the G_q/11_-PLC-PKC and MAPKs, but not through G_s_-cAMP-PKA pathway.

**Figure 4:**
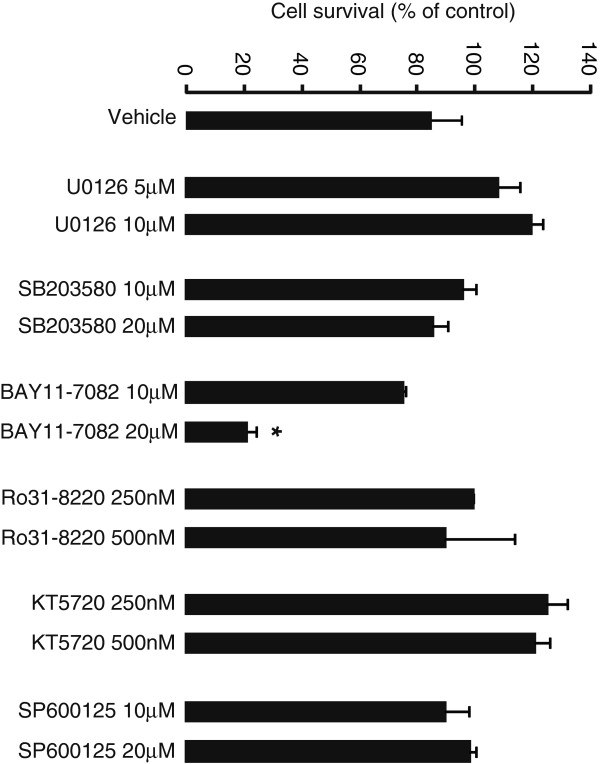
**Effect of signaling pathway inhibitors on survival of cultured astrocytes.** Primary cortical astrocytes were treated without or with different signaling pathway inhibitors used in this study (U 0126, 5 to 10 μM; SB 203580, 10 to 20 μM; BAY 11–7082, 10 to 20 μM; Ro 31–8220, 250 to 500nM; KT 5720, 250 to 500nM; SP 600125, 10 to 20 μM) for 24 hours and cell survival assessed by a colorimetric MTT assay. The optical densities were measured at 570 nm, with a 630 nm and blank correction. Data are normalized to percent of control (vehicle) and presented as Mean ± S.E.M of three independent experiments. *P* < 0.05, when compared to control.

In order to analyze the intracellular signaling pathways that couple A_2B_ receptor activity and LIF expression and release in astrocytes, various specific blockers of signaling routes were used. To determine the potential toxicity of these blockers, cultured astrocytes were incubated for 24 hours with the used and the doubled concentration of these blockers, and cellular survival was assessed by MTT assay. None of the used blockers at the appropriate concentration caused significant toxicity; the only blocker that negatively influenced astrocytic survival at the double concentration was the NF-κB inhibitor BAY 11-7082 (Figure 4).

**Figure 5:**
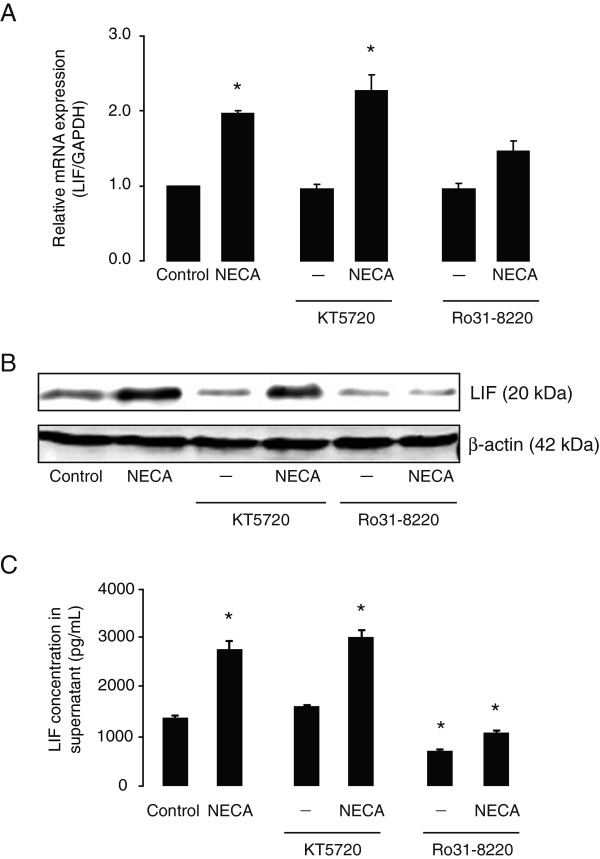
**NECA-induced leukemia inhibitory factor (LIF) expression and secretion levels in primary astrocytes are blocked by protein kinase C (PKC) inhibitor, but not by protein kinase A (PKA) inhibitor.** Primary cortical astrocytes were treated without or with NECA (1 μM) for 2 hours (for real-time PCR) or 4 hours (for Western blot and ELISA). Where indicated, astrocytes were pre-treated for 30 minutes with specific inhibitors of PKA (KT 5720, 250 nM) or PKC (Ro 31–8220, 250 nM), prior to NECA stimulation. **(A)** shows real-time PCR analysis of LIF gene expression (relative to *GAPDH*). Data are normalized to the control and presented as Mean ± SEM of three independent experiments. *P* < 0.05. **(B)** shows Western blot analysis to detect LIF protein levels. β-actin served as a loading control. **(C)** shows a representation of sandwich ELISA experiment performed to detect LIF content in supernatants of cultured astrocytes. Each bar corresponds to the mean concentration of LIF in triplicate samples; error bars indicate SEM. Observations were confirmed by repeating the experiments two additional times. *P* < 0.05. NECA, 5′-N-ethylcarboxamide.

Adenosine A_2B_ receptors are coupled to two types of G-proteins: G_s_ and G_q/11_[45]. Activation of G_s_ proteins stimulates cyclic AMP (cAMP) leading to PKA activation or Exchange Protein Activated by cAMP (EPAC) signaling pathways [46], whereas G_q/11_ proteins stimulate PKC *via* phospholipase C (PLC). In order to determine which pathway downstream of the A_2B_ receptor is responsible for NECA-induced LIF expression and release, we used specific inhibitors of PKA and PKC (KT 5720 and Ro 31–8220, respectively) [47]. Pre-incubation of astrocytes with KT 5720 (250 nM, added 30 minutes before NECA) did not affect NECA-induced LIF expression (both mRNA and proteins) (Figure 5A and 5B). However, pre-treatment with Ro 31-8220 (250 nM, added 30 minutes before NECA) reduced NECA-induced LIF mRNA (Figure 5A) as well as protein levels (Figure 5B). Consistently, Ro 31-8220, but not KT 5720, suppressed LIF content in the supernatant from NECA-treated astrocyte cultures (Figure 5C), indicating that adenosine A_2B_ receptor-mediated LIF expression and release from astrocytes require PKC, but not PKA, activation. 

### Basal and NECA-induced LIF expression and secretion levels in primary astrocytes are dependent on ERK1/2- and p38- but not on JNK-MAPK activation

**Figure 6:**
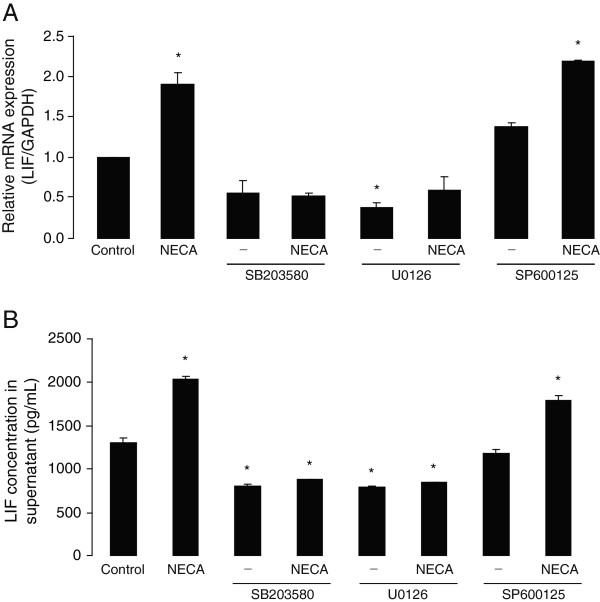
**Basal and NECA-induced leukemia inhibitory factor (LIF) expression and secretion levels in primary astrocytes are dependent on ERK1/2- and p38- but not on JNK-MAPK activation.** Primary cortical astrocytes were treated without or with NECA (1 μM) for 2 hours (for real-time PCR) or 4 hours (for ELISA). Where indicated, astrocytes were pre-treated for 2 hours with specific inhibitors of ERK1/2 (U 0126, 5 μM), p38 (SB 203580, 10 μM) or JNK (SP 600125, 10 μM) pathways, prior to NECA stimulation. **(A)** shows real-time PCR analysis of LIF gene expression (relative to *GAPDH*). Data are normalized to the control and presented as Mean ± SEM of three independent experiments. *P* < 0.05. **(B)** shows a representation of sandwich ELISA experiment performed to detect LIF content in supernatants of cultured astrocytes. Each bar corresponds to the mean concentration of LIF in triplicate samples; error bars indicate SEM. Observations were confirmed by repeating the experiments two additional times. *P* < 0.05. ERK, extracellular signal-regulated kinase; JNK, c-Jun N-terminal kinase; MAPK, mitogen-activated protein kinase; NECA, 5′-N-ethylcarboxamide.

Activation of the PKC pathway has been associated with effects mediated through mitogen-activated protein kinase (MAPK) signaling [48,49]. In order to determine the involvement of MAPKs in NECA-induced LIF expression and release, specific inhibitors of the three MAPK cascades: p38, extracellular signal-regulated kinase (ERK) 1/2, and c-Jun N-terminal kinase (JNK) (SB 203580, U 0126 and SP 600125, respectively), were used. It is shown here that pre-treatment (2 hours before NECA) of cultured astrocytes with SB 203580 (10 μM) and U 0126 (5 μM) significantly reduced basal as well as NECA-induced LIF mRNA expression (Figure 6A) and protein release (Figure 6B). On the other hand, pre-treatment with SP 600125 (10 μM, added 2 hours before NECA) affected neither basal, nor NECA-induced LIF mRNA expression or protein release (Figure 6A and 6B), suggesting that both p38 and ERK1/2, but not JNK, are important for basal, as well as NECA-induced, LIF expression and release in cultured astrocytes. 

### Basal and NECA-induced LIF expression and secretion levels in primary astrocytes are dependent on NF-κB activation

**Figure 7:**
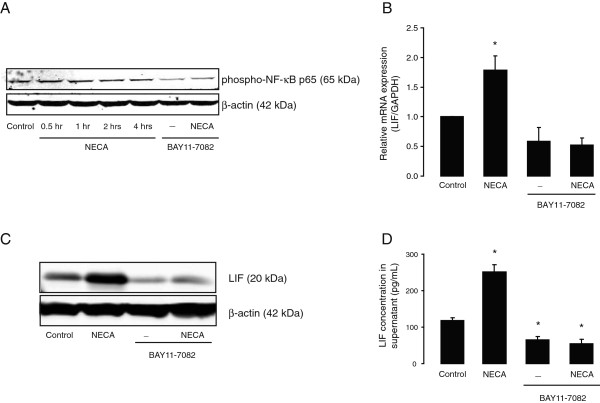
**Basal and NECA-induced leukemia inhibitory factor (LIF) expression and secretion levels in primary astrocytes are dependent on NF-**κ**B activation. (A)** shows Western blot analysis of primary cortical astrocytes treated without or with NECA (1 μM; for 0.5, 1, 2 and 4 hours) to detect phosphorylation at Ser536 of NF-κB p65 (RelA) proteins. Where indicated, cells were pre-treated with selective inhibitor of NF-κB (BAY 11–7082, 10 μM) for 2 hours prior to NECA stimulation. β-actin served as a loading control. Subsequently, astrocytes were treated without or with NECA (1 μM) for 2 hours (for real-time PCR) and 4 hours (for Western blot and ELISA), in presence or absence of BAY 11–7082 (10 μM, added 2 hours prior to NECA stimulation). **(B)** shows real-time PCR analysis of LIF gene expression (relative to *GAPDH*). Data are normalized to the control and presented as Mean ± SEM of three independent experiments. *P* < 0.05. **(C)** shows Western blot analysis to detect LIF protein levels. β-actin served as a loading control. **(D)** shows a representation of sandwich ELISA experiment performed to detect LIF content in supernatants of cultured astrocytes. Each bar corresponds to the mean concentration of LIF in triplicate samples; error bars indicate SEM. Observations were confirmed by repeating the experiments two additional times. *P* < 0.05. NECA, 5′-N-ethylcarboxamide.

IL-6 gene expression in cultured astrocytes is enhanced by NECA [36,38], similar to our present findings with LIF. Since NF-κB is a key transcription factor that regulates IL-6 gene expression [50,51], we wondered whether NECA-induced LIF gene expression is similarly regulated by NF-κB. Analysis of the mouse LIF promoter region (using Genomatix-MatInspector software (http://www.genomatix.de/)), identified multiple consensus binding sequences for NF-κB at the first 500 bp upstream to the transcription start site (data not shown). Treatment of cultured astrocytes with NECA (1 μM) for 0.5, 1, 2 and 4 hours, induced activation of NF-κB, which was detected by phosphorylation of NF-κB p65 subunit by Western blot (Figure 7A). In addition, the specific inhibitor of NF-κB activation, BAY 11-7082 (10 μM; added 2 hours before NECA) reduced NECA-induced phosphorylation of NF-κB p65 (Figure 7A) and significantly inhibited basal as well as NECA-induced LIF expression (both mRNA and protein) and release (Figure 7B–D), strongly indicating that LIF gene expression is regulated by NF-κB. 

### LIF secretion in primary astrocytes is constitutive and independent of NECA stimulation

**Figure 8:**
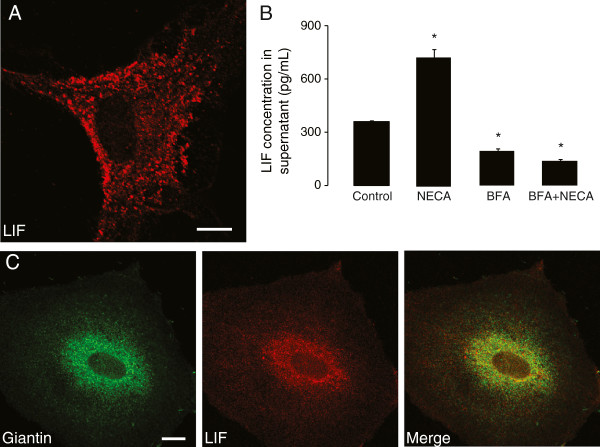
**Leukemia inhibitory factor (LIF) secretion in primary astrocytes is constitutive and independent of NECA stimulation. (A)** illustrates LIF immunocytochemistry in cultured cortical astrocytes, where LIF is found in vesicle-like structures throughout the cytoplasm. Scale bar corresponds to 10 μm. **(B)** shows complete co-localization of LIF with Giantin, a marker of Golgi apparatus, when cultured astrocytes were treated with Brefeldin A (BFA, 5 μg/mL for 1 hour). Scale bar corresponds to 10 μm. **(C)** shows a representation of sandwich ELISA experiment performed to detect LIF content in supernatants of cultured astrocytes that were treated without or with NECA (1 μM) for 4 hours. Where indicated, cells were pre-treated with BFA (5 μg/mL) for 1 hour prior to NECA stimulation. Each bar corresponds to the mean concentration of LIF in triplicate samples; error bars indicate SEM. Observations were confirmed by repeating the experiments two additional times. *P* < 0.05. NECA, 5′-N-ethylcarboxamide.

Supernatants from untreated astrocytes contained basal levels of LIF, suggesting that it could be constitutively released from these cells. Since we observed increased LIF concentrations in astrocyte supernatants after NECA stimulation, we investigated whether or not NECA played a direct role in LIF secretion. Thus, we blocked the early secretory pathway with Brefeldin A (BFA), a fungal metabolite that causes Golgi-derived proteins to accumulate in the endoplasmic reticulum [52]. Cultured astrocytes were pre-treated for 1 hour with BFA (5 μg/mL), followed by treatment without or with NECA (1 μM) for 1 or 4 hours (for immunocytochemistry and LIF-ELISA, respectively). In BFA-treated cells, all LIF-immunoreactivity co-localized with the Golgi marker Giantin (Figure 8B), compared to control conditions, where punctate LIF stainings could be observed all over the cytoplasm (Figure 8A). LIF levels in the supernatants from BFA-treated (5 μg/mL, for 5 hours) astrocyte cultures were significantly lower than that of the control (Figure 8C), implying that LIF is indeed constitutively secreted. Moreover, NECA treatment (1 μM, for 4 hours) did not stimulate LIF secretion in BFA-treated astrocytes (Figure 8C), further indicating that increased LIF levels in astrocytic culture supernatants after NECA stimulation require synthesis of new proteins and does not involve a ready-releasable post-Golgi reservoir of LIF. 

### LIF secretion in primary astrocytes is mediated through recycling endosomes

**Figure 9:**
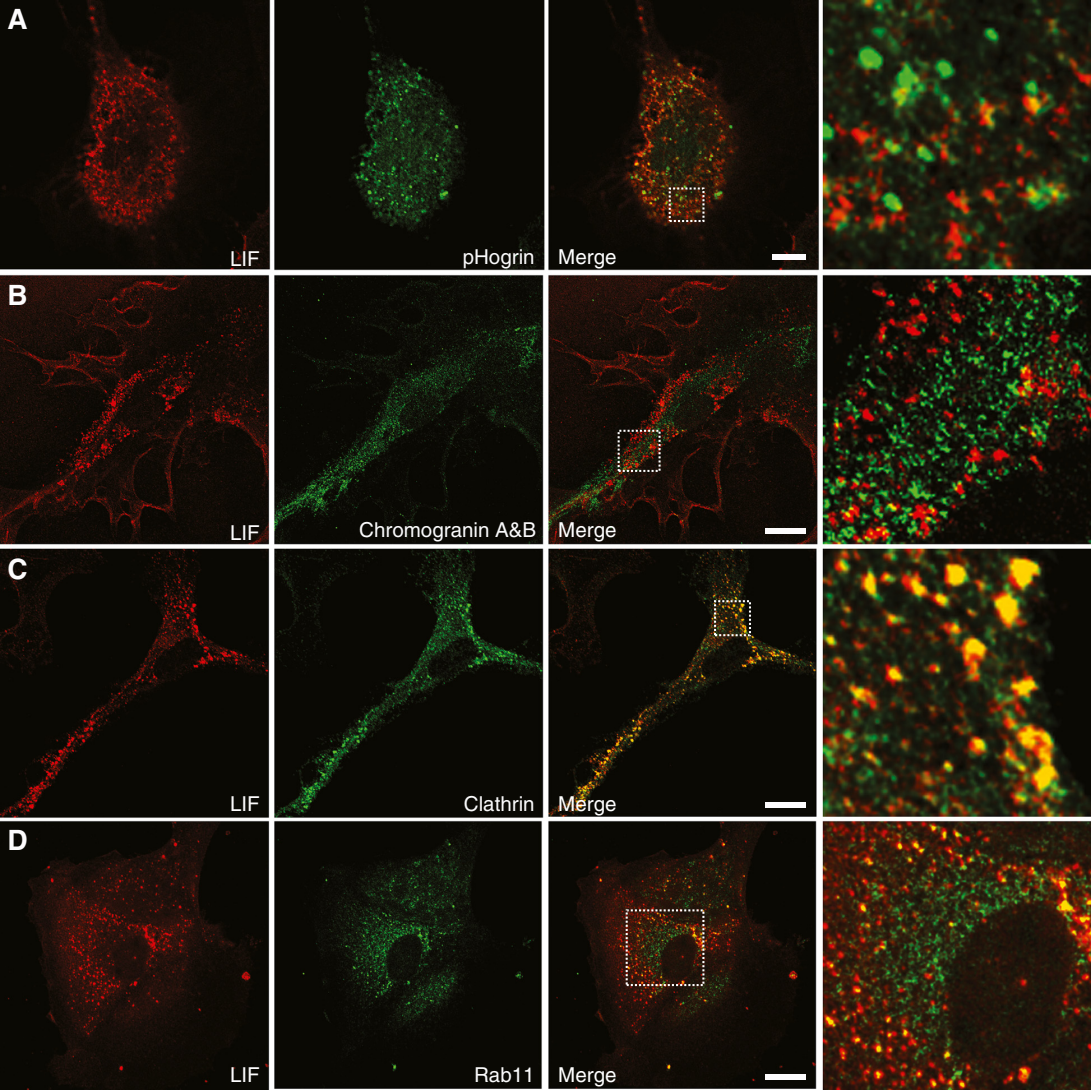
**Leukemia inhibitory factor (LIF) co-localizes with clathrin and Rab11 but not with large dense-core vesicle markers.** Immunostaining performed in cultured astrocytes revealed that LIF does not co-localize with pHogrin (**A**) and Chromogranin A & B (**B**), which are both markers for large dense-core vesicles. However, we observed a massive co-localization of LIF with clathrin (**C**) and a partial co-localization with Rab11 (**D**). Scale bars correspond to 10 μm.

We further investigated the type of organelles responsible for LIF release. Several reports have shown that cytokines such as IL-6 and transforming growth factor β (TGFβ) are secreted through specialized secretory granules called large dense-core vesicles (LDCV) [53,54]. However, when astrocytes were co-stained for LDCV markers such as Chromogranin A & B, or pHogrin and LIF, no co-localization was observed (Figure 9A and 9B). In contrast, co-localization between LIF and clathrin was observed (Figure 9C). Clathrin is a marker for endosomal vesicles and is sometimes used as a marker for constitutive release [55,56]. Furthermore, LIF partially co-localized with Rab11 (Figure 9D), which is a marker for recycling endosomes [57,58], suggesting that recycling endosomes, rather than LDCV, mediate secretion of LIF. 

### NECA-treated astrocytes induce LIF-mediated protection of cultured cortical neurons against excitotoxicity

**Figure 10:**
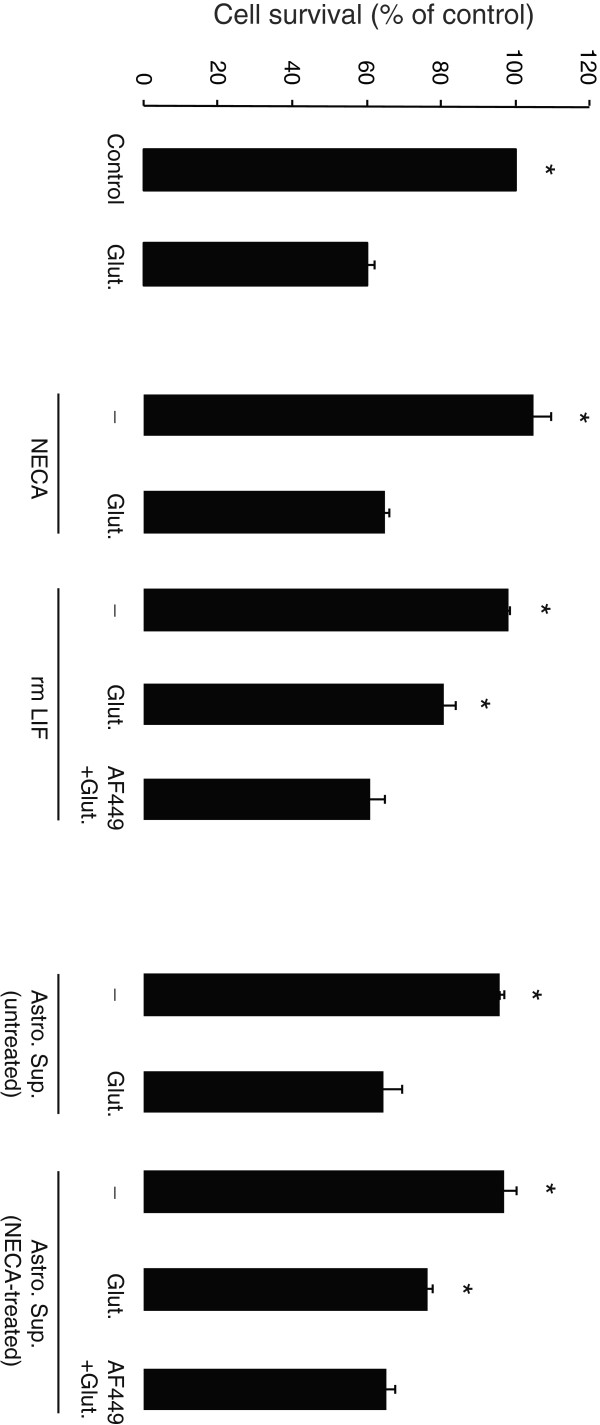
**Effect of untreated and NECA-treated astrocyte supernatants on survival of cultured cortical neurons against excitotoxicity.** Primary cortical neurons were treated without or with recombinant mouse Leukemia inhibitory factor (rmLIF, 0.1 ng/mL), NECA (1 μM) or astrocyte supernatants (untreated or treated with 1 μM NECA for 4 hours; diluted 1:1 in neuronal culture medium) for 24 hours. Where indicated, rmLIF and NECA-treated astrocyte supernatants were treated with an anti-LIF neutralizing antibody (AF449; 100 ng/mL for 1.5 hours) before applying them to cultured neurons. Subsequently, the neurons were treated without or with glutamate (Glut.; 50 μM for 1 hour) and cell survival assessed 24 hours after glutamate treatment by a colorimetric MTT assay. The optical densities were measured at 570 nm, with a 630 nm and blank correction. Data are normalized to percent of control and presented as Mean ± S.E.M of four independent experiments. *P* < 0.05, when compared to glutamate-treated condition. NECA, 5′-N-ethylcarboxamide.

We have previously shown that recombinant mouse LIF (rmLIF) protein protects mouse cortical neurons against excitotoxicity [13]. In order to understand whether NECA stimulation of astrocytes specifically would induce accumulation of neuroprotective LIF, astrocyte cultures were refreshed with new media shortly before NECA stimulation (1 μM for 4 hours) and the supernatant was collected. As shown in Figure 10, pre-treatment (1:1 dilution, for 24 hours) with supernatant from NECA-treated astrocytes significantly reduced the glutamate-induced cell death of cultured cortical neurons. A similar protective effect was observed by pre-treatment with rmLIF (0.1 ng/mL, for 24 hours) (Figure 10). Pre-treatment of the neurons (for 24 hours before application of glutamate) with NECA (1 μM) or supernatant from untreated astrocytes did not affect glutamate-induced neuronal cell death (Figure 10). We further investigated whether neuroprotection induced by NECA-treated astrocyte supernatant was mediated by LIF, by incubating the supernatants for 1.5 hours at 37°C with a LIF-neutralizing antibody (goat polyclonal anti-LIF, AF449, 100 ng/mL, R&D Systems, Oxford, UK) before applying to the neuronal cultures. The optimum concentration of LIF-neutralizing antibody was standardized by an efficiency test, performed according to manufacturer’s recommendations (data not shown). In addition, the effect of rmLIF protein treated with LIF-neutralizing antibody, on neuronal survival against glutamate, served as a control (Figure 10). Interestingly, LIF-neutralized supernatant from NECA-treated astrocyte cultures failed to protect neurons against glutamate (Figure 10), suggesting a direct neuroprotective mechanism of the endogenous LIF produced by astrocytes in response to NECA stimulation. 

## Discussion

We have previously shown that recombinant LIF protects neurons against glutamate-induced excitotoxicity [13]. In this study, we investigated the mechanism by which astrocytes produce and release LIF. Here we show that glutamate-induced neuronal excitotoxicity leads to adenosine receptor-mediated increase in LIF mRNA expression in cultured cortical astrocytes. We demonstrate that the upregulation of LIF mRNA and protein is adenosine A_2B_ receptor-dependent, and is mediated through G_q/11_-PLC-PKC-MAPK-NF-κB signaling pathways. We furthermore show that LIF is transiting through the Golgi and is found in recycling endosomes rather than in LDCV. Finally, LIF produced by astrocytes can protect neurons against excitotoxicity.

It has been known for more than a decade that astrocytes are the major source for LIF in the CNS [18,19,59,60]. However, the factors responsible for the regulation of LIF expression in these cells are still largely unknown. It is well known that stressed neurons release nucleotides such as ATP and adenosine [30,61]. Recently, it was demonstrated that astrocytes increase LIF production and release in response to ATP receptor stimulation [18]*.* In this study, the authors demonstrate that neurons during action potentials can secrete ATP, which triggers LIF production in astrocytes. This ATP-dependent upregulation of LIF by astrocytes is responsible for the promotion of oligodendrocyte-mediated myelination around neuronal axons. ATP is also known to be secreted by neurons during stressful conditions such as seizure, ischemia and hypoxia [26,27]. However, when we blocked adenosine receptors with the non-selective antagonist caffeine, or with specific A_2A_/A_2B_ receptor antagonists, the effect of glutamate-stressed neuronal supernatants on LIF expression in astrocytes was absent, suggesting that adenosine, but not ATP, is responsible for astrocytic LIF production during glutamate-induced neuronal stress. Thus, it might be hypothesized that depending on the CNS status, astrocytic LIF expression and secretion is differentially regulated; during normal neuronal activity and development ATP is involved whereas during neuronal insults, adenosine might enhance LIF secretion by astrocytes.

Several studies have demonstrated the involvement of adenosine A_2B_ receptors in the regulation of IL-6 expression in various cell types *in vitro*[38,47,48,62,63] as well as *in vivo*[64], suggesting that A_2B_ receptors might also be essential in the regulation of other IL-6-type cytokines. Our results show that adenosine-dependent LIF regulation is mediated through the A_2B_ receptor, since no increase in LIF expression was found in cultured astrocytes from A_2B_ receptor deficient mice. Instead NECA caused a down-regulation of LIF mRNA after 8 and 24 hours in these cells, indicating that knocking out A_2B_ receptors may have unmasked an inhibitory effect on LIF mRNA expression of an unidentified adenosine receptor. Whether or not this might explain the very short-lived effect of NECA on LIF mRNA expression in wild-type astrocytes is at the moment unclear and a subject of future investigations. We furthermore demonstrated that A_2B_-mediated LIF expression is dependent on the PKC, but not the PKA pathway. These data are in line with the study of Aloisi and colleagues, which demonstrated that LIF modulation by pro-inflammatory cytokines in human astrocytes was mediated through PKC activation [59]. Moreover, PKC has also been shown to be essential in IL-6 regulation [47,48,62,65], revealing a prominent role for PKC in the signaling pathway controlling LIF gene expression.

MAPKs have been reported to be involved in adenosine A_2B_ receptor-mediated regulation of IL-6 gene expression in astrocytoma cells [48]. In our experiments, both basal as well as NECA-induced LIF gene expression and release in cultured astrocytes were inhibited by specific inhibitors of p38 and ERK1/2, but not JNK-MAPKs. In line with our findings, it has been shown that LIF expression in Schwann cells is mediated through PKC pathway-induced ERK1/2 activation [49]. Furthermore, we show here that adenosine-dependent LIF expression in astrocytes is regulated through the NF-κB transcription factor. This observation is in line with several studies showing an NF-κB-dependent regulation of IL-6 gene by this transcription factor in several cell types [38,50,51,65,66]. It has been shown that NECA-induced NF-κB activation and the resultant IL-6 gene expression was abolished by inhibitors of MAPK pathways [65]. In our study, preliminary observations indicate that NECA-induced activation of the NF-κB pathway is reduced by selective inhibitors of p38 and ERK1/2 pathways (data not shown), suggesting that these pathways might play as upstream mediators in NF-κB-dependent LIF expression in astrocytes.

Recent evidence indicates that, depending on the cell type, different secretory pathways are employed for cytokine release [67]. For example, T cells use two different release mechanisms: IL-2 and IFN-γ are secreted at the immunological synapse whereas CCL3 and TNF-α are secreted multidirectionally, suggesting different secretory pathways [68]. In neurons or neuron-like cells, secretory granules called LDCVs are the organelles used for the selective secretion of IL-6, TGF-β2 and CCL21 [53,54,69]. The same organelles are also used in immune cells such as mast cells and neutrophils [67]. Here we show that LIF protein is transported through Golgi but its secretion by astrocytes is not mediated by secretory granules. Instead, LIF co-localizes with Rab11, a known marker of recycling endosomes [57,58]. Moreover, we observed a partial co-localization of LIF with clathrin, which also associates with recycling endosomes where it is implicated in protein sorting [56]. Recycling endosomes have now been shown to be responsible for cytokine secretion in several cell types. For example, IFN-γ and TNF-α secretion from natural killer cells require Rab11 [70]. Recycling endosomes are also responsible for the constitutive secretion of IL-6 and TNF-α in macrophages [71]. Further studies will be needed to better understand LIF sorting, trafficking and release by these vesicles.

Interestingly, our data indicate that LIF is constitutively released from astrocytes. Indeed constant levels of LIF were present in the supernatants of untreated astrocytes when measured by ELISA. Similar data were observed in human astrocyte cultures [59]. Whether this observation is representative of the physiological behavior of astrocytes *in vivo* or is due to the culture conditions remains to be determined. We further show that by blocking the early secretory pathway with BFA, the LIF concentration in the culture supernatant was not increased upon NECA stimulation. The inhibitory effect of BFA indicates that LIF passes through the Golgi prior to its secretion, and thus does not follow non-conventional secretory pathways that by-pass the Golgi and is typically insensitive to BFA, which has recently been reported to be used by other cytokines [72]. Importantly, the inhibitory effect of BFA suggests that NECA-stimulated release of LIF by astrocytes requires *de novo* LIF synthesis, and does not involve a ready-releasable post-Golgi pool of LIF.

It is now clear that one of the major roles of LIF is directed toward cell protection. Indeed, it has been shown that LIF is up regulated in astrocytes and neurons after cerebral ischemia [21] as well as in astrocytes after cortical brain injury [24], suggesting a role of LIF in neuronal repair or protection. In line with these data, treatment of rat with LIF prevented loss of motoneurons after peripheral nerve injury [73,74] and protection of retinal ganglia cells was compromised in LIF knock-out mice after lens injury [12]. Finally, LIF was shown to limit demyelination in an experimental autoimmune encephalomyelitis mouse model [16] and has become a prominent therapeutic candidate for multiple sclerosis [17]. We have previously shown that LIF can protect cortical as well as hippocampal neurons against glutamate-induced excitotoxicity [13]. Here we show that LIF coming from the supernatant of NECA-treated astrocytes has the same protective effect. Indeed, astrocytes produce several other cytokines and neurotrophic factors including IL-6, NGF, brain-derived neurotrophic factor, neurotrophin-3, S-100β protein and TGFβ [75], that might help neurons to cope with excitotoxic stress. Accordingly, conditioned media from astrocyte cultures protected cortical neurons against glutamate (data not shown). In order to confine the neuroprotective effect of astrocytic factors that are released in response to NECA treatment, we had to refresh astrocyte culture medium prior to NECA treatment and testing supernatant on glutamate-stressed neurons. This, together with LIF neutralization, indicates that LIF produced by astrocytes after adenosine receptor stimulation is necessary to witness neuronal protection. Our results provide further evidence for a role of adenosine in neuronal protection. Indeed, it has been shown that adenosine can protect neurons during hypoxia [76,77], ischemia [78,79] and excessive neuronal activity [80,81]. This adenosine protection is often mediated through the A_1_ receptor subtype [82-84], but here we show that an indirect protection of adenosine through the stimulation of A_2B_ receptor on astrocytes leading to LIF upregulation exists. This A_2B_ receptor activation might be related to an anti-inflammatory process as observed previously by others [85-87].

## Conclusions

We demonstrate a protective role of LIF against glutamate neurotoxicity and we provide clear evidence that adenosine is required for an increased production of LIF by astrocytes. These data further confirm a neuroprotective role of adenosine in the brain.

## Abbreviations

A2B KO, A2B receptor knock-out; BFA, Brefeldin A; bp, base pairs; ChAT, choline acetyltransferase; CNS, central nervous system; CNTF, ciliary neurotrophic factor; CT-1, cardiotrophin-1; DMEM, Dulbecco’s modified Eagle’s medium; DMSO, dimethyl sulfoxide; EDTA, ethylenediaminetetraacetic acid; EPAC, Exchange Protein Activated by cAMP; ERK, extracellular signal-regulated kinase; FCS, fetal calf serum; HBSS, Hank’s balanced salt solution; HEPES, hydroxyethyl piperazineethanesulfonic acid; HRP, horseradish peroxidase; IL, interleukin; IFN, interferon; JNK, c-Jun N-terminal kinase; LDCV, large dense-core vesicles; LIF, leukemia inhibitory factor; LME, L-leucine methyl ester; MAP2, Microtubule-associated protein 2; MAPK, mitogen-activated protein kinase; NECA, 5′-N-ethylcarboxamide; NGF, nerve growth factor; NNT-1, novel neurotrophin-1; NT, nuclear transcription factor; OBB, Odyssey™ Blocking Buffer; OSM, oncostatin M; PBS, phosphate-buffered saline; PBS-T, phosphate-buffered saline plus 0.1 % Tween 20; PKA, protein kinase A; PKC, protein kinase C; PLC, phospholipase C; PVDF, polyvinylidene fluoride; qPCR, real-time PCR; rmLIF, recombinant mouse LIF; TGFβ, transforming growth factor β.

## Competing interests

The authors declare that they have no competing interests.

## Authors’ contributions

SM performed the majority of experiments including cell culturing, ELISA and Western blotting. JV did the immunofluorescent part of the study as well as all the microscopy and statistical analysis. Both SM and JV were involved in the redaction of the manuscript. EW did the qPCR experiments. JB took part in ELISA experiments. CHS performed the toxicity and the A_2B_ KO experiments. SCDI and PB were both involved in the secretion part of the study. They gave precious antibodies, were essential in the design of the experiments and participated actively in writing the manuscript. HWGMB and KPHB were both involved in the conception and design of the study as well as in the manuscript redaction. All authors read and approved the final manuscript.
